# The Role of Semantic Context in Early Morphological Processing

**DOI:** 10.3389/fpsyg.2017.00991

**Published:** 2017-06-15

**Authors:** Caroline M. Whiting, Richard G. Cowley, Mirjana Bozic

**Affiliations:** ^1^Institute of Neuroscience and Psychology, University of GlasgowGlasgow, United Kingdom; ^2^Department of Psychology, University of CambridgeCambridge, United Kingdom

**Keywords:** morphology, semantic context, morphological decomposition, masked priming, visual word recognition

## Abstract

There is extensive evidence pointing to an early, automatic segmentation of written words into their constituent units (*farm-er, wit-ness*); however, less is known about the potential role of contextual information in modulating this analysis. We adapted the standard masked priming paradigm to include an overt semantic prime in order to examine whether semantic context influences morpho-orthographic segmentation of complex words. In particular, we asked how the context will affect processing of semantically opaque forms (*witness*), where the embedded stem (*wit*) is incompatible with the meaning of the whole form. Results showed no masked priming facilitation for opaque forms in the presence of a semantic prime, indicating that context can influence early morphological analysis. Priming was found for both semantically transparent and opaque forms (*farmer-farm, witness-wit*) when there was no semantically-related context, consistent with the literature and an account positing early blind segmentation. These findings provide an important update to the long-standing debate on early morphological processing in written word recognition.

## Introduction

A substantial body of evidence has revealed that complex words undergo an early, blind, and automatic segmentation into their constituent parts (Rastle et al., 2004; Longtin and Meunier, 2005; Marslen-Wilson et al., 2008; McCormick et al., 2008; Rastle and Davis, 2008). This process of bottom-up, stimulus-driven word segmentation highlights the critical role of morphological structure in lexical access, whereby written word forms are automatically broken down into meaningful subunits, even when these subunits are not compatible with the meaning of the whole form (e.g., *corner* is not someone who corns). Thus, *farmer* facilitates the recognition of *farm, witness* facilitates the recognition of *wit*, but *spinach* does not facilitate the recognition of *spin* since the final letters (*-ach*) do not form a meaningful suffix in English. At a later processing stage, however, only *farmer* still facilitates the recognition of *farm* (but *witness* does not facilitate *wit*), suggesting that only semantically transparent words, such as *farmer* retain morphemic structure at the level of central lexical representations (Marslen-Wilson et al., 1994).

The point at which lexical semantics begins to exert influence on morphological processing is still a matter of debate. According to the form-before-meaning account, morpho-semantic properties of complex words determine the organization of central lexical representations, but not the process of lexical access (Marslen-Wilson et al., 1994; Rastle et al., 2000; Meunier and Longtin, 2007). Recent evidence has further supported the claim that semantics does not play a role in early morpho-orthographic processing, given that blind segmentation is triggered for opaque forms even when the suffix conveys no regular meaning (e.g., *-er* in *ponder*; Beyersmann et al., 2016). Only later stages of word recognition are argued to be semantically driven, when the meaning of the whole form is accessible, and makes the interpretation of *witness* as containing a stem *wit* and suffix *-ness* invalid. This is supported by evidence from paradigms in which the prime is consciously perceived, such as cross-modal priming (Meunier and Longtin, 2007), where only semantically transparent forms (*farmer*) significantly facilitate the processing of their constituent stem (*farm*). An alternative account argues that semantic information is accessed early during morphological processing, in parallel with orthographic information (Diependaele et al., 2005; Feldman et al., 2009, 2015). In this account, masked priming effects are argued to show a graded effect depending on the strength of the relationship between stem and whole-form meaning—hence, larger effects for transparent forms and weaker effects for opaque form.

Here we approach the issue of semantic influences on morphological processing from a different perspective, asking about the role of semantic context in early morpho-orthographic segmentation. Can this bottom-up, stimulus-driven process of segmenting potential stems and suffixes be modulated by contextual constraints? The majority of evidence for morpho-orthographic segmentation has come from studies of words in isolation (*farmer* priming *farm*), and may therefore to some extent reflect this specific experimental setup. We investigated the effects of context to test how language processing takes place in an environment which is closer to reading in real life, where words are embedded into a wider semantic setting.

Extensive neuroimaging evidence has demonstrated top-down modulation of early processing in domains, such as speech perception (Davis and Johnsrude, 2007) and low-level visual processing (Gazzaley et al., 2005; Rauss et al., 2011). Furthermore, language comprehension and production models have long posited interactivity (McClelland and Rumelhart, 1981), suggesting a key role for contextual information and prior knowledge on bottom-up processes. Given the strong claims concerning blind, data-driven analysis of morpho-orthographic structure, it is therefore important to consider whether this bottom-up process could be constrained by contextual information.

The existing morphological processing literature offers limited evidence. One recent eye-tracking study (Amenta et al., 2015) used a property specific to Italian to examine how sentential context interacts with morpho-orthographic segmentation. In this study, derived Italian words were embedded in sentences that altered word meaning toward either a transparent or opaque interpretation. The authors report stem frequency effects for both transparent and opaque meanings on first fixation duration, a measure of early processing. This suggests that all forms are initially segmented regardless of the context. However, the effect on first fixation duration was facilitatory for transparent meanings and inhibitory for opaque meanings, indicating that semantic information for the stem and suffix is available, and results in an incongruent interpretation for opaque forms.

The effect of sentence context on morphological processing has also been investigated in a cross-modal priming study of German particle verbs (Zwitserlood et al., 2005). These results demonstrated differing priming effects for semantically transparent (e.g., *meebrengen*, bring along—related to the stem *brengen*, bring) and semantically opaque verbs (e.g., *ombrengen*, exterminate—unrelated to the embedded stem *brengen*) when primed by the meaning of the stem (*brengen*). Transparent forms showed priming but opaque forms did not, which was interpreted as evidence for separable representations for opaque forms and their constituent stems at the conceptual level. However, it is not clear whether early stages of morphological processing were modulated by the context—before the relationship between the prime and target was consciously processed—which was not directly investigated in this study.

Our goal in this study was to incorporate a semantic context within the masked priming paradigm, to assess if there is an interaction between the context and the analysis of morpho-orthographic structure. In a standard masked priming study, morpho-orthographic segmentation of the prime is indexed by the facilitation of target word recognition (as compared to an unrelated control). When presented in isolation as masked primes, both semantically transparent and opaque forms (*farmer, witness*) are segmented, resulting in facilitated recognition for the stem targets (Rastle et al., 2004; Marslen-Wilson et al., 2008; McCormick et al., 2008). The critical aspect of our design is the inclusion of a visible prime that preceded the masked prime, which can provide information about the whole-form meaning of the masked prime: e.g., a masked prime *witness* preceded by a fully visible word *jury* (see Table 1). We then manipulated how the visible prime was related to the masked prime. A visible prime that is semantically unrelated to the masked prime (e.g., *tulip–witness–wit*) is not expected to interfere with the morpho-orthographic segmentation of the masked prime. As such, any resultant facilitation of the target would be due to the relationship between the masked prime and target (*witness–wit*). This context allows us to compare the current results with the existing literature on morpho-orthographic segmentation.

**Table 1:** Example experimental stimuli in the three prime contexts and three conditions.

		**Condition**								
		**Transparent**			**Opaque**			**Stem Only**		
		**Sem. prime**	**Mask prime**	**Target**	**Sem. prime**	**Mask prime**	**Target**	**Sem. prime**	**Mask prime**	**Target**
	+**Semantic** +**Mask**	crop	farmer	FARM	jury	witness	WIT	lettuce	spinach	SPIN
**Context**	**–Semantic** +**Mask**	smooth	farmer	FARM	tulip	witness	WIT	wool	spinach	SPIN
	**Control**	smooth	mixer	FARM	tulip	jetty	WIT	wool	monkey	SPIN

The primary focus of interest, however, was the context where the visible prime was semantically related to the masked prime. For semantically transparent words like *farmer*, a preceding visible prime like *crop* is consistent with both the whole-form meaning of the word *farmer* and its morphological constituents. However, for semantically opaque forms, such as *witness* preceded by a semantically related word *jury*, the meaning of the stem *wit* is incompatible with the semantic context of *jury*. Based on a form-before-meaning account (Rastle et al., 2004; Crepaldi et al., 2010), all words containing a stem and a suffix are segmented in a bottom-up manner; thus, *farmer* will prime *farm* and *witness* will prime *wit*. However, given a semantic context that biases the interpretation toward the whole-form meaning (*witness* → *jury/crime/court*, etc.), it is unclear whether *witness* will still be segmented based on a top-down modulation of bottom-up processing.

There are two possible outcomes here: blind morpho-orthographic segmentation could take place regardless of the context—purely based on the bottom-up input—thus both *farmer* and *witness* will prime their respective stems even when the semantic context is incongruent with the semantically opaque stem (*wit*). Alternatively, the context could interact with bottom-up, form-based processing, resulting in a reduced or absent masked priming effect for semantically opaque forms (*witness-wit*), but still showing priming for semantically transparent forms (*farmer-farm*). This second prediction is not necessarily inconsistent with a form-before-meaning account, but it would require a re-interpretation of previous results by allowing for contextual modulations on morphological processing when words are not processed in isolation.

In summary, the present study included three conditions and three prime contexts in a modified masked priming design, where an overt semantic prime was followed by a masked prime and a target. The three conditions were: (1) semantically transparent forms (*farmer-farm*), (2) semantically opaque forms (*witness-wit*), and (3) stem-only forms (*spinach-spin*). They were presented in three prime contexts: (1) with an overt prime semantically related to the masked prime (+Semantic +Mask; e.g., *jury-witness-wit*); (2) with an overt prime semantically unrelated to the masked prime (–Semantic +Mask; e.g., *tulip-witness-wit*); and (3) a baseline control context, where neither the overt nor the masked prime was related to the target (Control; e.g., *tulip-jetty-wit*). This final baseline context is not expected to trigger any facilitation of the processing of the target word, thus serving as an overall control.

In the context where the masked prime is preceded by a semantically unrelated prime (Context 2; –Semantic +Mask; e.g., *tulip-witness-wit*), we expect to see priming in the transparent and opaque conditions but not in the stem-only condition, consistent with the previous literature (e.g., Rastle et al., 2004). The critical context is Context 1, where the masked prime is preceded by a semantically related word (+Semantic +Mask). Here, the primary focus is on the semantically opaque condition (e.g., *jury-witness-wit*), with the key question being whether opaque forms like *witness* will still undergo morpho-orthographic segmentation into *wit* + *-ness* (and therefore facilitate the recognition of the target *wit*) when the segmented meaning is inconsistent with the context. If *witness* no longer primes *wit* in the context of a semantically related prime *jury*, this would suggest that external semantic variables can influence early morphological processing.

Finally, the current design included an additional control condition to test for the direct effects of semantic context on the target. This is because the semantic prime is related in meaning to both the masked prime and the target for semantically transparent forms in Context 1 (+Semantic +Mask; e.g., *crop-farmer-farm*); thus, both primes may play a role in facilitating recognition of the target. To disentangle the contribution of the two primes, this control condition included word triplets in which only the semantic prime was related to the target (+Semantic –Mask; e.g., *crow-paler-hawk*). If the semantically transparent condition with two related primes (+Semantic +Mask; e.g., *crop-farmer-farm*) shows faster recognition times compared to the +Semantic –Mask control context, this will further confirm that the masked prime is modulating processing of the target, and that our paradigm is indeed tapping into the intended early stages of visual word recognition.

## Methods

### Materials and design

We included three experimental conditions that co-varied the presence/absence of a potential suffix, as well as the relationship between the stem and suffix. These included: (1) semantically transparent forms (e.g., *farmer*), which contain both a stem and suffix (*farm* + *-er*), and where the meaning of the stem is related to the meaning of the whole form; (2) semantically opaque forms (e.g., *witness*), which contain both a stem and suffix (*wit* + *-ness*), but the meaning of the stem (*wit*) is unrelated to the meaning of the whole form; and (3) stem-only forms (e.g., *spinach*), where there is an embedded stem (*spin*) but the remaining letters (-*ach*) do not form a potential suffix in English. In all conditions, the embedded stem appeared as the target for lexical decision and the whole form appeared as the masked prime.

To create different priming contexts, a further prime was included which was presented overtly prior to the masked prime. This overt prime was either semantically related or unrelated to the meaning of the masked prime, as measured by Latent Semantic Analysis (http://lsa.colorado.edu/). To create these contexts, we used the LSA Near Neighbors function to pair each masked prime in the three conditions (*farmer, witness, spinach*) with a semantically related form that did not overlap in the first letter (in order to remove any potential priming effects due to orthographic overlap) and was not morphologically complex. These sets of semantic prime-masked prime-target triplets (e.g., *crop-farmer-FARM*) formed the +Semantic +Mask prime context. Secondly, to form the –Semantic +Mask prime context (e.g., *smooth-farmer-FARM*), where the masked prime is still related to the target but the semantic and the masked primes are unrelated, the semantic primes were randomized within condition and paired with an unrelated masked prime. Finally, the masked primes were randomized within condition and paired with an unrelated target to create the Control prime context (e.g., *smooth-mixer-FARM*). Thus, every target appeared in three contexts: (1) with the overt prime related to the masked prime, and the masked prime related to the target; (2) with the overt prime unrelated to the masked prime and the masked prime related to the target; and (3) with both overt and masked primes semantically unrelated to the target (see Table 1 for the full set of conditions and prime contexts).

A total of 30 items were selected for each condition (transparent, opaque, and stem only), and conditions were matched on target length, wordform frequency, neighborhood (N) size and bigram frequency; masked prime length, wordform frequency and % orthographic overlap between target and masked prime; and semantic prime length, wordform frequency, neighborhood size, bigram frequency, and semantic relatedness (Latent Semantic Analysis) between the masked prime and semantic prime (all *ps* > 0.1 in a one-way ANOVA across conditions; see Table 2). Wordform frequency measures were taken from the CELEX database (Baayen et al., 1995), and bigram frequency and N size measures were taken from the MCWord database (Medler and Binder, 2005). Stimuli are contained in Table A1.

**Table 2:** Stimulus properties across test conditions (mean values).

		**Condition**			
		**Transparent**	**Opaque**	**Stem Only**	**Semantic Prime Only**
**Target**	Length	4.23	4.00	4.03	4.40
	Frequency	41.26	26.67	21.07	36.25
	N size	8.03	9.90	8.57	8.2
	Bigram Frequency	1113.76	1399.93	1167.53	1199.93
	Target/Masked Prime Relatedness (LSA)	0.48	0.09	0.05	0.10
	Target/Semantic Prime Relatedness (LSA)	0.42	0.11	0.05	0.56
**Masked Prime**	Length	6.23	5.93	6.47	6.63
	Frequency	7.17	13.58	7.98	12.05
	N size	2.53	3.23	0.77	2.07
	Bigram Frequency	1033.67	1137.16	608.37	1058.29
	Target/Masked Prime % Overlap	0.69	0.68	0.63	0.69
	Masked Prime/Semantic Prime Relatedness (LSA)	0.48	0.49	0.54	0.11
**Semantic Prime**	Length	5.47	5.07	4.90	4.73
	Frequency	41.5	67.67	56.65	47.06
	N size	4.63	6.07	4.43	5.87
	Bigram Frequency	1076.79	1868.01	1755.04	1430.91

*Far right-hand column indicates semantic prime only condition (+Semantic –Mask) included as an additional test for the target-semantic prime relationship. The transparent condition (crop-farmer-FARM) and the semantic prime only condition (crow-paler-HAWK) are the two instances where the target and semantic prime are related in meaning*.

A further set of 30 word targets were paired with a related semantic prime and an unrelated masked prime to create a +Semantic –Mask condition (e.g., *hawk-paler-CROW*). This condition served two purposes: firstly, to investigate the priming effect due solely to the relationship between the semantic prime and the target, which are both overt. As discussed earlier, this is relevant because the transparent condition in the +Semantic +Mask prime context contains a semantic prime (*crop*) that is related to both the masked prime (*farmer*) and the target (*farm*), and it is not possible to determine straightforwardly whether any priming effect is simply due to the influence of the semantic prime, and not the masked prime. Secondly, this allowed us to balance out the stimuli set such that the number of trials where the overt prime and target are related was 1/3 of all word trials. Previous evidence has demonstrated that the proportion of semantically related trials can affect the size of the priming effect (Neely et al., 1989; Hutchison et al., 2001). Thus, we wanted to ensure that the number of semantic primes was large enough to produce a robust semantic priming effect. All items in the +Semantic –Mask condition were matched to the remaining test items on length, frequency, N size, and bigram frequency of the target; length and frequency of the masked prime; % orthographic overlap between masked prime and target, and length and frequency of the semantic prime (all *ps* > 0.1). The +Semantic –Mask stimuli are contained in Table A2.

A set of 120 pseudowords were created using the ARC Non-word Database (Rastle et al., 2002) that were orthographically and phonologically legal sequences and matched to the real word targets based on length, neighborhood size and bigram frequency. Each pseudoword target was then randomly assigned two word primes (e.g., *hollow-traced-SWAUK*), to generate a triplet similar to the experimental sets but ending in a pseudoword target. This allowed us to have an equal number of word and non-word responses for the lexical decision task, and crucially, the non-word trials were not predictable from the primes.

The nine semantic prime-masked prime-target sets (3 conditions × 3 prime contexts) were pseudo-randomized into 3 versions (10 sets from each condition in each version) such that each target appeared only once in each version. Each participant saw one version, ensuring there was no repetition of the targets. The +Semantic –Mask condition (*hawk-paler-CROW*) and the pseudowords (*daze-busy-HIF*) did not have repeating targets, and thus were presented to all participants. Each participant therefore saw a total of 240 trials.

### Procedure

**Figure 1:**
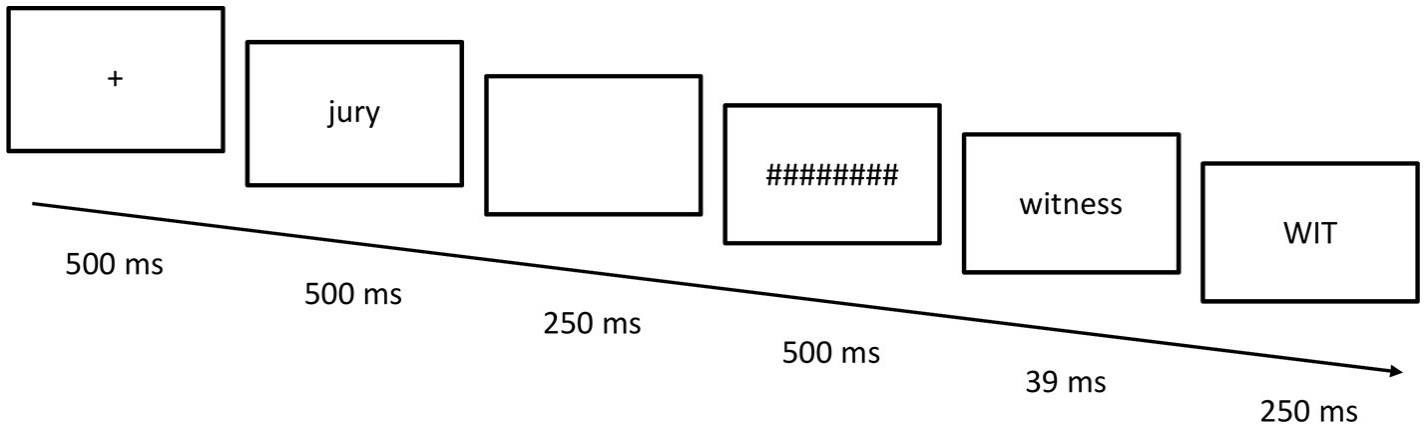
Timeline of a single trial.

Subjects were told that they would see a word followed by hash marks and a letter string in uppercase, and should decide as quickly and accurately as possible whether the uppercase string was a real word in English or not. They were not told about the existence of masked primes. A crosshair was displayed for 500 ms in the center of the screen followed by the semantic prime for 500 ms, a blank screen for 250 ms, the forward mask (hash marks) for 500 ms, a masked prime for 39 ms, and a target for 250 ms (see Figure 1). Targets were in upper case and both semantic and masked primes were in lower case to avoid any visual overlap between prime and target. Participants received 20 trials as practice before the experiment began. Stimulus presentation and data recording were controlled by the E-Prime 2.0 software (Psychology Software Tools, Pittsburgh, PA). The experiment lasted approximately 15 min.

### Subjects

A total of 77 subjects took part in the experiment (age range: 18–27, mean: 21; 52 female). One subject was subsequently removed from the analyses as they were not a native speaker. All remaining subjects were right handed native British English speakers, with no reading or learning difficulties. Written informed consent was obtained from all participants, and they were paid for their participation.

## Results

All errors (5.1% of trials) and all time-outs (defined as responses longer than 1,500 ms; 0.02% of trials) were removed. One item from the stem-only condition (*heave*) was excluded because of error rates over 50%.

### Reaction time analysis

The mean reaction time (RT) for word targets was 556 ms, and 632 ms for pseudowords. To analyse the data we used linear mixed-effect models (Baayen et al., 2008) as implemented in the lme4 R package (Bates et al., 2015), with subjects and items as crossed random effects. By-subject random slopes for prime context and condition were evaluated individually using likelihood ratio tests. Prime context contributed significantly to the model fit, but condition did not and was removed from the analysis.

The dependent variable was log-transformed reaction time, and the main predictors were prime context (3 levels: +Semantic +Mask, –Semantic +Mask, Control), condition (3 levels: transparent, opaque, stem only), and the interaction between prime context and condition. We also included version, target length, masked prime length, semantic prime length, % orthographic overlap between target and masked prime, target N size, and log-transformed wordform frequencies of target, masked prime, and semantic prime as predictors. We used the Satterthwaite approximation for degrees of freedom (Satterthwaite, 1946) as implemented in the lmerTest R package (Kuznetsova et al., 2015). To arrive at the best-fitting model, we used the step function in the lmerTest R package on the full model containing the three prime contexts, three conditions, and all fixed and random effects. Only the prime context, condition, version, and log-transformed target frequency, along with subjects and items, emerged as significant predictors, and are included in subsequent analyses reported below. Significant *p*-values are reported at *p* < 0.05.

In the overall 3 (prime context) × 3 (condition) analysis, there was a significant effect of prime context [*F*_(2, 119)_ = 25.29, *p* < 0.0001], and a significant effect of condition [*F*_(2, 77)_ = 11.26, *p* < 0.0001]. The effect of version was not significant (*F* < 1). The interaction between prime context and condition was significant [*F*_(4, 6156)_ = 6.70, *p* < 0.0001], indicating that the context had a differing effect across the three conditions. Target frequency was also a significant predictor of RT [*F*_(1, 77)_ = 31.10, *p* < 0.0001]. There was no significant interaction between prime context, condition and version (*F* < 1), therefore all following analyses will collapse across version.

**Figure 2:**
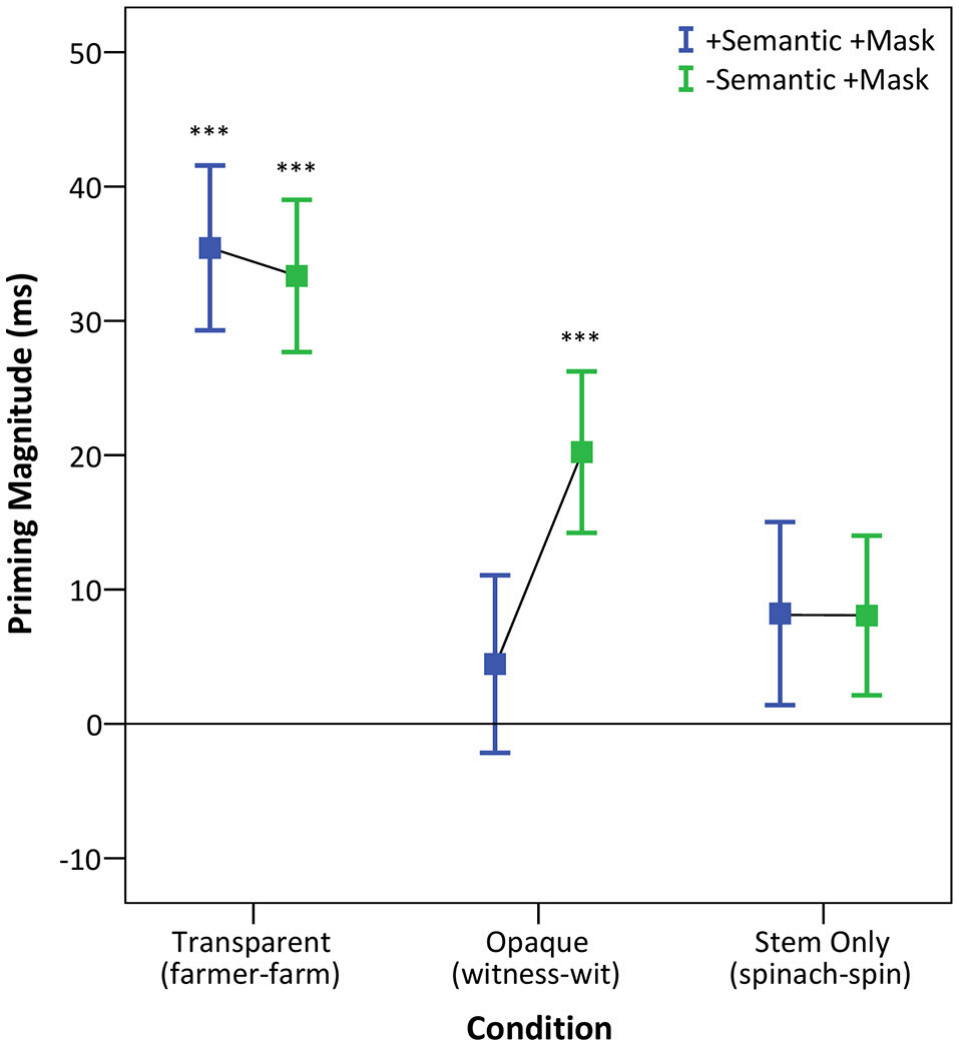
Average priming effect in milliseconds (mean ± standard error), defined as the difference between the unrelated prime type context (Control) and the other prime contexts. ^***^Indicates significant at *p* < 0.001.

Given the significant overall interaction between prime context and condition, we first performed a series of planned comparisons to examine how context affected priming across the three conditions (for full results see Figure 2 and Table 3).

**Table 3:** Reactions times (RTs; mean and standard deviation) and error rates (%) for each condition in each context, and the overall priming effect compared to baseline (Control).

	**Condition**					
	**Transparent (*****farmer-FARM*****)**		**Opaque (*****witness-WIT*****)**		**Stem Only (*****spinach-SPIN*****)**	
	**RT ms (s.d.)**	**Errors %**	**RT ms (s.d.)**	**Errors %**	**RT ms (s.d.)**	**Errors %**
+**Semantic** +**Mask**	519 (80)	1.3	558 (83)	6.1	576 (85)	6.6
**–Semantic** +**Mask**	521 (73)	1.3	542 (76)	3.8	576 (84)	6.8
**Control**	554 (69)	3.7	562 (70)	7.5	585 (72)	9.1
**Priming effect in** +**Semantic** +**Mask context**	35^***^		**4**		**8**	
**Priming effect in –Semantic** +**Mask context**	33^***^		20^***^		**8**	

*** *Indicates significant at p < 0.001*.

We began by examining masked priming effects across the three conditions in the –Semantic + Mask context, comparing it to the Control context. This was done to assess how our results relate to the literature on standard masked morphological priming (Rastle et al., 2004; Longtin and Meunier, 2005; Marslen-Wilson et al., 2008), which does not use semantic context to modulate the processing of the masked prime. Based on these previous studies, we predicted that the related masked prime should generate a priming effect for both transparent and opaque forms (*farmer-FARM; witness-WIT*) as compared to the Control baseline, but not for the stem-only condition (*spinach-SPIN*). This resulted in a 2 (prime context) × 3 (condition) design, where the masked prime was either related or unrelated to the target—equivalent to previous masked priming studies.

Results showed a significant effect of prime context [*F*_(1, 272)_ = 48.70, *p* < 0.0001] reflecting significant facilitation due to the presence of a masked prime, and a significant effect of condition [*F*_(2, 82)_ = 7.43, *p* < 0.005]. The interaction between prime context and condition was significant [*F*_(2, 4100)_ = 6.58, *p* < 0.005], indicating that the masked priming effect varied across the three conditions. Specifically, we saw significant priming in the transparent (*p* < 0.0001) and the opaque condition (*p* < 0.0005), but not in the stem only condition (*p* > 0.05), consistent with the existing masked priming literature.

The next analysis tested priming effects across the three conditions in the +Semantic +Mask context, comparing it to the Control context, resulting in a 2 (prime context) × 3 (condition) design. Results showed a significant effect of prime context [*F*_(1, 73)_ = 29.58, *p* < 0.0001], a significant effect of condition [*F*_(2, 81)_ = 5.89, *p* < 0.005], and a significant prime context by condition interaction [*F*_(2, 4015)_ = 11.01, *p* < 0.0001]. In this context, significant priming emerged only in the transparent condition (*p* < 0.0001). Neither opaque not the stem only condition showed significant priming (both *ps* > 0.05).

The results presented so far suggest that context strongly affects priming in the morphologically related transparent and opaque conditions. To test this explicitly, the third set of planned comparisons involved a direct comparison of priming effects in the transparent and opaque conditions across the three contexts. This resulted in a 3 (prime context) × 2 (condition) design. Results showed a significant effect of prime context [*F*_(2, 90)_ = 29.18, *p* < 0.0001], a significant effect of condition [*F*_(1, 54)_ = 4.15, *p* < 0.05], and a significant interaction between prime context and condition [*F*_(2, 4161)_ = 8.65, *p* < 0.0005].

To unpack this interaction, we examined the priming effects in the transparent and opaque conditions separately: firstly, for the masked prime only context (–Semantic +Mask vs. Control), and secondly, for the two prime context (+Semantic +Mask vs. Control) in a 2 (prime context) × 2 (condition) design. For the masked prime context, there was no significant interaction between prime context and condition [*F*_(1, 2716)_ = 3.73, *p* > 0.05], suggesting that the masked priming effect did not differ between transparent and opaque forms. However, the interaction was significant for the +Semantic +Mask vs. Control context [*F*_(1, 2761)_ = 16.74, *p* < 0.0001], demonstrating that in the presence of a related semantic prime, transparent and opaque forms show differential priming effects.

Finally, we directly compared priming effects across contexts in each of the three conditions separately. For transparent forms (*farmer*), there was a significant effect of prime context [*F*_(2, 127)_ = 32.27, *p* < 0.0001]. The +Semantic +Mask context produced a significant priming effect compared to the Control baseline (*p* < 0.0001). The presence of a related masked prime only (–Semantic +Mask context) also produced a significant priming effect compared to the Control baseline (*p* < 0.0001). There was no significant difference between priming effects in the two primed contexts (+Semantic +Mask vs. –Semantic +Mask; *p* > 0.05).

For opaque forms (*witness*), there was a significant effect of prime context [*F*_(2, 141)_ = 7.77, *p* < 0.001]. The presence of related semantic and masked primes (+Semantic +Mask context) did not produce a significant priming effect compared to the baseline Control context (*p* > 0.05). However, the presence of a related masked prime (–Semantic +Mask context) produced a significant priming effect compared to the Control baseline (*p* < 0.0005). Furthermore, there was a significant difference (*p* < 0.05) between the amount of priming in the context of two related primes (+Semantic +Mask) and masked prime only (–Semantic +Mask).

For stem only forms (*spinach*), there was no significant effect of prime context [*F*_(2, 131)_ = 2.01, *p* > 0.05], indicating that RTs were not modulated by the presence of the different prime contexts.

The final RT analysis was the contrast of the two conditions where the overt semantic prime and target were related in meaning. This involved the transparent forms with two related primes (+Semantic +Mask; *crop-farmer-FARM*) and the semantic prime only condition (+Semantic –Mask; *hawk-paler-CROW*), which are the only conditions in the study where the semantic prime and the target are related. This contrast allows us to assess the amount of priming that may be due to the semantic relationship between the overt semantic prime and the target, regardless of the masked prime. Due to the fact that each participant saw 10 transparent items with two related primes (*crop-farmer-FARM*), but 30 items from the semantic prime only condition (*hawk-paler-CROW*) we randomly divided the semantic prime condition into three sets of 10 items in order to equalize the items in each condition. To ensure that this did not result in an uneven distribution of items across the three sets, we tested the transparent forms against all three sets of semantic prime items separately, as well as together. Mean RT, standard deviation, and error rate for the three lists are as follows: (1) *RT* = 558 ms, std. dev. = 133, error = 7.8%; (2) *RT* = 553 ms, std. dev. = 126, error = 4.7%; (3) *RT* = 570 ms, std. dev. = 138, error = 8.4%.

The transparent condition with two related primes (*crop-farmer-FARM*) showed significantly faster RTs compared to all three semantic prime lists [list 1: *F*_(1, 33)_ = 31.99, *p* < 0.0001; list 2: *F*_(1, 33)_ = 8.11, *p* < 0.01; list 3: *F*_(1, 36)_ = 17.22, *p* < 0.0005]. Combining all three lists in one analysis, the transparent condition with two primes (*crop-farmer-FARM*) again showed significantly faster RTs compared to the semantic prime condition [*hawk-paler-*CROW; *F*_(1, 71)_ = 21.82, *p* < 0.0001], further suggesting that the presence of masked primes indeed drives the observed effects. Log-transformed target frequency was a significant predictor for two out of the three semantic lists [*F*_(1, 36)_ = 3.58, *p* > 0.05; *F*_(1, 35)_ = 12.96, *p* < 0.001; *F*_(1, 35)_ = 16.57, *p* < 0.0005, respectively], as well as for the overall analysis using all items in the semantic prime condition [*F*_(1, 55)_ = 21.65, *p* < 0.0001].

### Error analysis

In the error analysis, we first examined the overall effects and the interactions as in the RT analysis, which included prime context (3 levels: +Semantic +Mask, –Semantic +Mask, Control) and condition (3 levels: transparent, opaque, stem only). For the errors, there was a significant effect of prime context [*F*_(2, 233)_ = 8.13, *p* < 0.0005], reflecting the higher number of errors in the Control (unprimed) context. There was, however, no significant effect of condition [*F*_(2, 86)_ = 1.53, *p* > 0.05], and the interaction between prime context and condition was also not significant (*F* < 1). Following this lack of explanatory interactions, no further analyses of error data were done.

## Discussion

In this study we aimed to test the possible interaction between top-down contextual information and bottom-up morphological analysis. Previous masked priming studies have demonstrated that words are automatically segmented into their constituent parts based on the presence of a stem and a suffix (Longtin and Meunier, 2005; Marslen-Wilson et al., 2008; McCormick et al., 2008; Rastle and Davis, 2008; Beyersmann et al., 2016). However, it is not known whether contextual information can modulate this data-driven segmentation and bias processing toward the whole-form meaning of a complex word.

To test this, we constructed an adapted masked priming design, where an overt semantic prime preceded the masked prime. Semantic priming is a robust effect showing faster and more accurate responses to a target if it is preceded by a semantically related word (Neely, 1991), which can modulate target reading times even at short prime durations (Sereno and Rayner, 1992). Here we focused on using the semantic prime to modulate processing of the masked prime rather than the target. By biasing the interpretation of the masked prime toward the whole-form meaning—the standard role of semantic context in natural language usage—we could assess if early morphological processing could be altered by previous contextual information. The key question was whether the presence of such a context would modulate the amount of morpho-orthographic priming between the masked prime and the target, particularly for semantically opaque pairs like *witness-wit*—in other words, would *witness* still prime the stem *wit* when they are preceded by the semantic context of *jury*?

To ground our results in the existing literature, we first established that complex words undergo an automatic morpho-orthographic segmentation when presented without an interfering context. To this end we tested priming effects in the context where the masked prime was related to the target but the semantic prime was unrelated to the masked prime (–Semantic +Mask), compared to the baseline (Control). Our results revealed a significant interaction between prime context and condition, showing that both semantically transparent and opaque words (*farmer, witness*) significantly primed their stems (*farm, wit*), but that stem-only forms, such as *spinach* did not prime their stems (*spin*). Crucially, there was no significant difference between the masked priming effect for transparent and opaque forms. This confirms that the presence of a potential stem and suffix is sufficient for decomposition when there is no informative context for the whole-form meaning, consistent with the existing masked priming literature.

The addition of a semantic context, where the semantic prime is related to the masked prime (+Semantic +Mask; *crop-farmer-FARM, jury-witness-WIT*), revealed a significant interaction between prime context and condition. Only semantically transparent forms (*crop-farmer-FARM*) showed significant priming in this context. Semantically opaque forms, on the other hand, did not show a priming effect when presented in the context of a semantically related overt prime (*jury-witness-WIT*), and the difference between the amount of priming for transparent and the opaque forms was also significant. This absence of priming for opaque forms suggests that morpho-orthographic segmentation can be modulated by the external contextual environment. Stem-only forms (*spinach-spin*) did not show significant priming in any context, as predicted.

A set of further comparisons directly contrasted morphological priming effects across the three contexts. These data showed that there was no significant difference between the amount of priming for morphologically transparent forms in the context of two primes and a masked prime only (35 and 33 ms, respectively). In contrast, the amount of priming for morphologically opaque forms in the masked prime only context (–Semantic +Mask) was significantly different from that seen in the two prime context (+Semantic +Mask; 20 and 4 ms, respectively), further confirming that external contextual environment modulates morpho-orthographic segmentation for opaque words only.

With the use of this adapted masked priming design, it is important to consider the possible effect of the direct relationship between the overt semantic prime and the target, since both are fully visible. This is especially true in the context where the overt prime is semantically related to the masked prime (+Semantic +Mask), and the masked prime is a transparent form (*farmer*). In this context, the semantic prime (*crop*) is also related to the target meaning (*farm*). This is inherent to semantically transparent words: if the semantic prime is related to the masked prime then it will necessarily be related to the target. Therefore, we cannot rule out that the priming effect seen for the transparent forms in a semantically related context (*crop-farmer-FARM*) is due to priming from the semantic prime to the target. It is important to note that this only holds for transparent forms in the semantically related context; this is not present in any other condition or context of interest.

To address this issue, we included a control condition in which the semantic prime was related to the target but the masked prime was unrelated (+Semantic –Mask; e.g., *hawk-paler-CROW*). This allowed us to assess facilitation due to the overt relationship between the semantic prime and target, irrespective of the masked prime. We found significantly faster RTs in the +Semantic +Mask context (*crop-farmer-FARM*) compared to the control semantic condition (+Semantic –Mask; e.g., *hawk-paler-CROW*), suggesting that our effects are indeed driven by the processing of the masked prime. Further evidence that the masked prime is playing a key role in this study is demonstrated by the significant priming effect for transparent and opaque forms with an unrelated semantic prime (*smooth-farmer-FARM; tulip-witness-WIT*). In both of these cases, it is only the masked prime that could facilitate the target.

The finding that contextual information modulates morpho-orthographic segmentation for opaque words like *witness*, but does not affect priming for transparent words like *farmer*, appears at first to contradict the form-before-meaning account (Rastle and Davis, 2008). If semantic transparency modulates the amount of masked morphological priming, this could be interpreted as evidence for early morpho-semantic processing, rather than purely morpho-orthographic segmentation. Such interpretation would be more consistent with the form-with-meaning account (Feldman et al., 2009), in which meaning plays an early role in the processing of complex words, and semantic similarity between the masked prime and target determines the size of the priming effect. It is however difficult to position our results fully within either the form-before-meaning or the form-with-meaning account, as they both focus on the role of the word's internal morpho-semantic structure, and neither makes explicit predictions about contextual effects in early morphological analysis.

Whether or not the semantic makeup of an isolated complex word influences its morpho-orthographic segmentation from the very onset, our data unambiguously show that all words with a potential stem and a suffix undergo this automatic analysis—as illustrated by significant and statistically comparable priming for both semantically transparent and semantically opaque words (*farmer, witness*) in the –Semantic +Mask context, where the masked prime is related to the target (*farmer-FARM, witness-WIT*). Critically, our results also make clear that external contextual information can modulate this process by biasing the interpretation toward the whole-form meaning (*witness* as opposed to *wit*).

One way of accounting for these results is to hypothesize that this reflects rapid integration of semantic information following morpho-orthographic segmentation, similar to the interpretation offered by Amenta et al. (2015). As described earlier, this eye-tracking study embedded derived Italian words into sentences that altered their meanings toward either a transparent or opaque interpretation. Early effects of frequency of the embedded stem were seen for both transparent and opaque meanings, which were facilitatory for transparent meanings and inhibitory for opaque meanings. The authors suggested that all potentially complex forms are automatically decomposed regardless of semantic transparency, but that the meanings of decomposed stems and suffixes are accessed early. This is consistent with evidence from the fast priming paradigm concerning rapid activation of semantics during reading more generally (Sereno and Rayner, 1992). Whilst our results can be accommodated within this hypothesis, they cannot provide a definitive verification, as they leave open another possible interpretation of the locus of the effects.

According to this alternative explanation, the observed reduction of priming for *witness-WIT* in the context of overtly presented form *jury* reflects the way these forms are represented at the level of central lexical representations, i.e., in the mental lexicon. A large body of evidence from behavioral and neuroimaging studies suggests that opaque words like *witness* are represented as “whole forms,” defined as a separate lexical entry with no internal representation of morphemic structure (Bozic et al., 2013a,b). In these cases, the onset-embedded stem like *wit* constitutes a second, different representation. Data further show that this second representation acts as a strong competitor, delaying the recognition of the derived form in a manner similar to the cohort competition effects (Marslen-Wilson, 1987). Hence, the presentation of an overt semantically related prime *jury* would facilitate access to the stored whole-word representation of *witness*. As a result, the whole form *witness* will become a stronger competitor for the separately represented and semantically unrelated target *wit*, and morphological priming between them will be weakened or eliminated. Comparable data have been seen in many morphological priming studies, where overtly presented opaque words elicit no priming for their targets (Marslen-Wilson et al., 1994; Rastle et al., 2000).

Due to the very nature of the current (behavioral) data, we cannot pinpoint the exact processing level at which the overt semantic prime like *jury* modulates the processing of the masked prime *witness*. Thus, it is difficult to conclude whether the observed results reflect early effects of semantics on morpho-orthographic segmentation of complex words (preventing the incorrect segmentation of *witness* into *wit* + *-ness)*, or a consequence of semantic priming between the stored representations of *jury* and *witness* at the level of central lexical representations (increasing competition between the whole form and embedded stem target). Regardless of which of the two interpretations better accounts for our data however, we show that top-down semantic context can interfere with early morphological analysis of complex words. This further supports the findings about the key role for contextual information and prior knowledge on bottom-up processes, shown across a variety of cognitive domains (Gazzaley et al., 2005; Davis and Johnsrude, 2007).

An important issue to address in future studies is how morphological segmentation is modulated by different types of contexts. In this study, we primed the whole-form meaning using a semantic prime, but further research could use stronger constraints, such as full sentences with high cloze probability, where we might see further reduction in masked priming for opaque forms. An additional question to pursue is how priming is affected by contexts that prime aspects beyond whole-form meaning—for instance, what would happen if *wheat* primed *corner*, where the embedded stem (*corn*) is now the congruent context? To provide a fuller account of the interaction between bottom-up and top-down processes, magnetoencephalography (MEG) and electroencephalography (EEG) evidence would be integral for tracking both where and when morphological effects emerge in differing contexts.

To conclude, we have provided novel evidence on the role of semantic context in modulating early morphological processing. The presence of a contextual cue to the meaning of the whole form eliminated the priming effect for semantically opaque forms, such that *witness* no longer primed *wit*. However, our findings also revealed that blind morpho-orthographic segmentation is triggered in the absence of context—consistent with the form-before-meaning account—with significant masked priming effects for both semantically transparent and opaque forms. The results of this study point to an interaction between top-down information provided by the context and bottom-up analysis during early stages of visual word processing. With the current design, we cannot determine the exact nature of this interaction—whether automatic segmentation is blocked by the semantic context, or whether the top-down semantic information interacts with the resulting stem and suffix following segmentation. Further studies are needed in order to disentangle possible interpretations of findings, but the current results open up an important discussion about the role of contextual information on stimulus-driven processing during early stages of word processing.

## Ethics statement

This study was carried out in accordance with the recommendations of the Cambridge Psychology Research Ethics Committee with written informed consent from all subjects. All subjects gave written informed consent in accordance with the Declaration of Helsinki. The protocol was approved by the Cambridge Psychology Research Ethics Committee.

## Author contributions

CW, RC, and MB designed the study, acquired and analyzed the data, and wrote the manuscript.

### Conflict of interest statement

The authors declare that the research was conducted in the absence of any commercial or financial relationships that could be construed as a potential conflict of interest.

## Appendix

**Table A1:** Stimuli for three test conditions.

**Transparent**			**Opaque**			**Stem Only**		
**Semantic prime**	**Masked prime**	**Target**	**Semantic prime**	**Masked prime**	**Target**	**Semantic prime**	**Masked prime**	**Target**
steam	boiler	boil	flag	banner	ban	ice	arctic	arc
plane	bomber	bomb	neck	belly	bell	thief	bandit	band
summit	climber	climb	wine	brandy	brand	church	basilica	basil
rain	cloudy	cloud	weed	clover	clove	sketch	cartoon	cart
smooth	creamy	cream	aluminum	copper	cop	theater	costume	cost
weird	creepy	creep	biscuit	cracker	crack	slang	dialect	dial
fantasy	dreamer	dream	rude	cranky	crank	magic	dragon	drag
pleasure	enjoyable	enjoy	volcano	crater	crate	art	easel	ease
crop	farmer	farm	cabinet	drawer	draw	atom	electron	elect
damage	faulty	fault	witch	fairy	fair	orange	ginger	gin
soft	fluffy	fluff	mane	filly	fill	language	grammar	gram
damp	gloomy	gloom	twinkle	flicker	flick	music	harmony	harm
accuse	guilty	guilt	tulip	flower	flow	whale	harpoon	harp
celebrate	joyful	joy	beef	gravy	grave	pray	heaven	heave
pencil	marker	mark	chisel	hammer	ham	fire	inferno	infer
kitchen	mixer	mix	satire	irony	iron	lion	monkey	monk
silt	muddy	mud	harbor	jetty	jet	fork	napkin	nap
heal	painless	pain	somber	listless	list	bed	pillow	pill
red	pinkish	pink	hotel	lobby	lob	clam	plankton	plank
saddle	rider	ride	coin	penny	pen	sermon	pulpit	pulp
steal	robber	rob	think	ponder	pond	team	rugby	rug
sprint	runner	run	law	punish	pun	awake	slumber	slum
mood	sadness	sad	dish	saucer	sauce	lettuce	spinach	spin
purchase	seller	sell	thin	skinny	skin	hospital	surgeon	surge
afraid	shaky	shake	foot	sneaker	sneak	kilt	tartan	tart
fear	shameful	shame	autumn	summer	sum	bus	taxi	tax
gloss	silky	silk	war	treaty	treat	ant	termite	term
tune	singer	sing	fade	vanish	van	wool	textile	text
hurricane	stormy	storm	stroll	wander	wand	drink	tipsy	tip
pool	swimmer	swim	jury	witness	wit	culprit	villain	villa

**Table A2:** Stimuli for Semantic Prime Only (+Semantic –Mask) condition.

**Semantic Prime Only**		
**Semantic prime**	**Masked prime**	**Target**
prize	grassy	award
hay	sleepy	barn
trout	murky	fish
hornet	construction	bee
admit	worker	blame
job	dancer	boss
tent	viewer	camp
star	graceful	comet
hawk	paler	crow
soap	glider	dirt
brake	learner	drive
construct	mourner	build
fence	windy	gate
pale	thirsty	gaunt
joint	sweetness	hinge
smile	fruitless	hug
joke	driver	laugh
harvest	sculptor	maize
yard	teacher	porch
wheat	hunter	rice
task	wrecker	skill
frost	cheerful	sleet
warm	honesty	sun
bone	weaver	spine
chair	worthy	stool
fruit	visitor	sweet
visit	bulky	trip
blouse	dusty	vest
shirt	chilly	wear
cry	voter	wail
